# A Large-Scale Multispecialty Evaluation of Web-Based Simulation in Medical Microbiology Laboratory Education: Randomized Controlled Trial

**DOI:** 10.2196/72495

**Published:** 2025-07-30

**Authors:** Lei Xu, Xichuan Deng, Tingting Chen, Nan Lu, Yuran Wang, Jia Liu, Yanan Guo, Zeng Tu, Yuxin Nie, Yeganeh Hosseini, Yonglin He

**Affiliations:** 1 Department of Pathogenic Biology, School of Basic Medicine, Chongqing Medical University, 1 Medical College Road, Yuzhong District, Chongqing, China, 86 2368485288; 2 Pathogen Biology and Immunology Laboratory, Experimental Teaching and Management Center, Chongqing Medical University, Chongqing, China; 3 The Second Clinical College, Chongqing Medical University, Chongqing, China; 4 College of International Education, Chongqing Medical University, Chongqing, China

**Keywords:** medical education, virtual simulation, pathogenic cocci, blended learning, multispecialty evaluation, laboratory safety, cost-effectiveness

## Abstract

**Background:**

Traditional laboratory teaching of pathogenic cocci faces challenges in biosafety and standardization across medical specialties. While virtual simulation shows promise, evidence from large-scale, multidisciplinary studies remains limited.

**Objective:**

The study aims to evaluate the effectiveness of integrating virtual simulation with traditional laboratory practice in enhancing medical microbiology education, focusing on the identification of biosafety level 2 pathogenic cocci. The study assessed improvements in student performance, theoretical understanding, laboratory safety, and overall satisfaction, while achieving standardization and cost reduction across multiple medical specialties.

**Methods:**

This randomized controlled trial involved 1282 medical students from 9 specialties. The experimental group (n=653) received virtual simulation training—featuring interactivity and intelligent feedback—prior to traditional laboratory practice, while the control group (n=629) did not receive such training. Our virtual system focused on biosafety level 2 pathogenic cocci identification with dynamic specimen generation.

**Results:**

The experimental group showed significantly improved performance across specialties (*P*<.05 for each specialty), particularly in clinical medicine, in which the experimental group score was 89.88 (SD 13.09) and the control group score was 68.34 (SD 17.23; *P*<.001). The students reported that virtual simulation enhanced their theoretical understanding (1268/1282, 98.9%) and laboratory safety (1164/1282, 90.8%) while helping them achieve standardization (790/1282, 61.6%,) and cost reduction (957/1282, 74.6%). Overall student satisfaction reached 97.2% (1246/1282), with distinct learning patterns observed across specialties. The test scores were significantly higher in the experimental group, with a mean of 80.82 (SD 17.10), compared to the control group, with a mean of 67.45 (SD 16.81).

**Conclusions:**

This large-scale study demonstrates that integrating virtual simulation with traditional methods effectively enhances medical microbiology education, providing a standardized, safe, and cost-effective approach for teaching high-risk pathogenic experiments.

## Introduction

Pathogenic cocci remain significant pathogens in clinical practice, and experiments that include these microorganisms are crucial for medical education [1-3]. The increasing prevalence of antibiotic resistance and the inherent biosafety risks associated with handling pathogenic microorganisms present substantial challenges in educational settings [4-6]. Traditional laboratory teaching, while essential, faces limitations in ensuring consistent safety standards and providing standardized learning experiences across diverse medical specialties. Virtual simulation technology has emerged as a transformative tool in medical education [7-10]. In microbiology education specifically, this technology offers unique advantages in visualizing microscopic processes and safely simulating high-risk procedures [11,12]. Recent studies have demonstrated its potential in enhancing spatial understanding and mastery of physiological concepts [13-15]. However, most existing research has focused on single-specialty applications or small-scale implementations, leaving a significant knowledge gap regarding the effectiveness of virtual simulations across multiple medical disciplines [16-18]. To address these challenges, our teaching team developed a comprehensive system called Virtual Simulation Experiment for Pathogenic Cocci in Pus Specimens. This innovative system aims to revolutionize teaching on the subject of pathogenic cocci through 3 key features: dynamic specimen generation, integrated biosafety level 2 (BSL-2) safety protocols, and real-time performance tracking.

This study addresses critical questions in medical laboratory education. Specifically, this study aims to evaluate the effectiveness of virtual simulation across 9 medical specialties, representing one of the largest multidisciplinary investigations in this field. We seek to assess the impact on learning outcomes, safety enhancement, and cost-effectiveness in teaching related to pathogenic cocci experiments, while analyzing specialty-specific learning patterns and their implications for customizing virtual simulation approaches. By addressing these objectives, this research provides comprehensive insights into the integration of virtual simulation in medical microbiology education.

## Methods

### Virtual Simulation System

#### Experimental Content

Based on Kern’s 6-step curriculum development model [19], for the pathogenic cocci virtual simulation teaching system constructed for this study, we first identified core issues through teaching evaluation. We specifically identified a lack of standardization in BSL-2 pathogen handling and inadequacies in cocci identification skills (ie, problem identification). By analyzing biosafety standards and the needs of the teaching faculty, we established a hierarchical competency framework covering Gram staining, culture isolation, biochemical testing, and integrated diagnosis (ie, needs assessment). The system content strictly aligned with 3 key teaching objectives: the basic modules included standardized operating procedures compliant with aseptic techniques (objective formulation); the intermediate modules integrated a dynamic specimen generation system to enable diversified training (educational strategies); and the advanced modules incorporated an embedded assessment system to monitor diagnostic decision-making capabilities in real time (implementation and evaluation). All experimental procedures are embedded with BSL-2 protection protocols, and the precision of the virtual simulation meets the operational standards of BSL-2 laboratories (quality control during implementation).

#### System Features

The design of this virtual simulation platform fully embodies the closed-loop concept of the Kern model: interactive virtual microscopy technology addresses the pain point of limited specimen types in traditional teaching (problem orientation); the real-time operation feedback system corresponds to the error-correction needs in the hierarchical teaching objectives (objective alignment); and the automated assessment module not only enables immediate evaluation of operational accuracy (formative assessment) but also generates personalized learning curves, providing data support for continuous curriculum improvement (summative assessment). Compared with traditional experimental methods, the platform, through intelligent error-correction guidance and a virtual consumables system (optimization of educational strategies), ensures full coverage of operational standards (needs response) while reducing teaching costs (verification of implementation effectiveness), thus completely realizing a closed-loop curriculum development process from problem identification to effect evaluation.

### Study Design and Implementation

**Figure 1.**
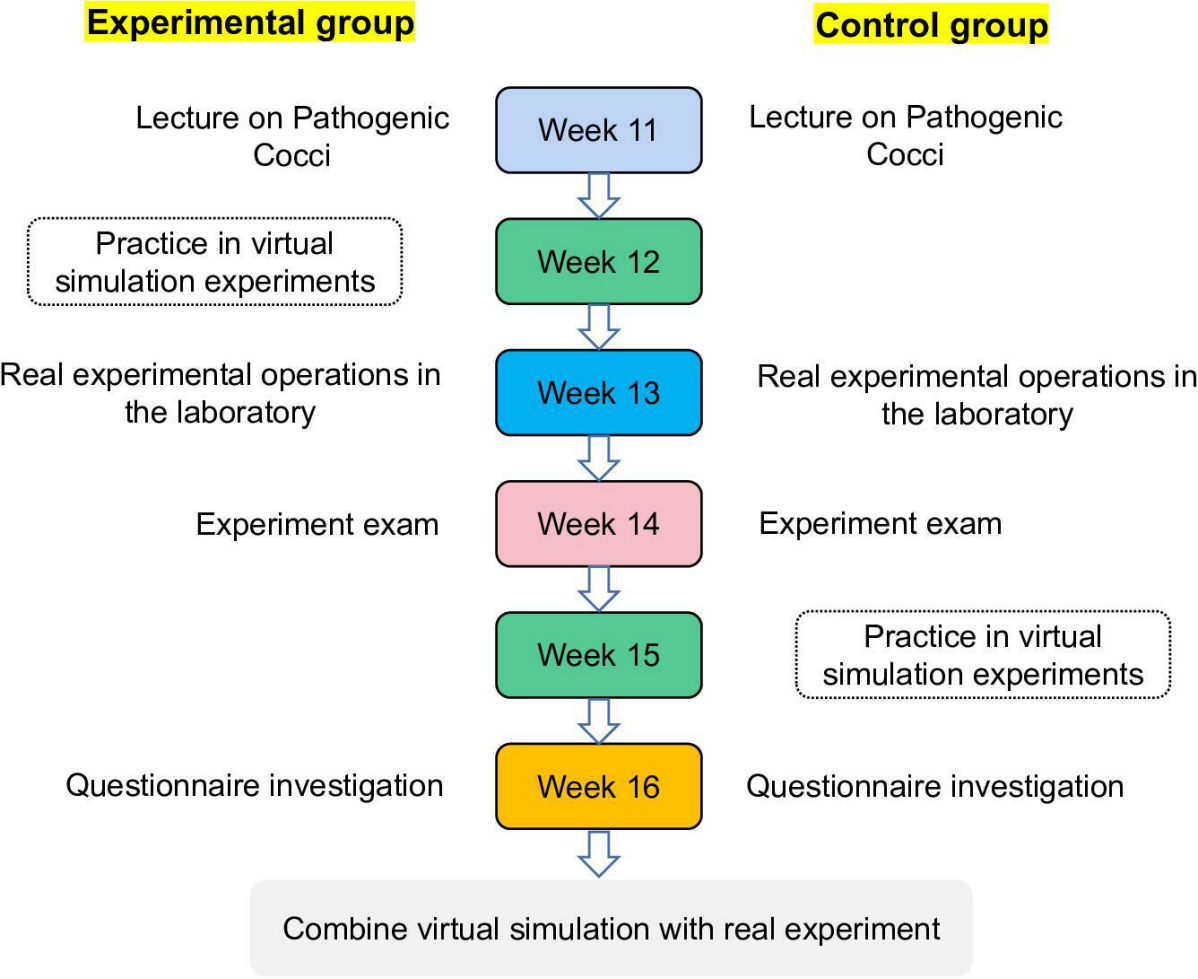
Course schedule.

This study used a randomized controlled trial design with a mixed methods evaluation spanning from September 2023 to January 2024. A total of 1282 medical students from 9 specialties (clinical medicine, traditional Chinese medicine, pediatrics, nursing, medical imaging, preventive medicine, clinical pharmacy, acupuncture, moxibustion, and traditional Chinese pharmacy) participated in the study, with equal, random assignment to experimental (n=653) and control (n=629) groups. The teaching implementation followed a systematic schedule across 6 weeks (detailed in Figure 1), integrating theoretical lectures, virtual simulation exercises, practical laboratory sessions, and comprehensive assessments of both groups. This design enabled direct comparison of learning effectiveness between traditional and virtual simulation–enhanced teaching approaches.

### Teaching Process

The implementation of this teaching system integrated both virtual simulation and traditional laboratory approaches. The teaching process followed a systematic structure comprising preclass preparation, in-class activities, and postclass assessment. All participating students received standardized learning materials and safety protocols, with the experimental group using the virtual simulation platform under instructor guidance while the control group followed traditional laboratory teaching methods. Both groups engaged in comprehensive learning activities designed to achieve the specified learning objectives, with emphasis on laboratory safety awareness and standardized experimental procedures.

### Data Collection

A comprehensive evaluation framework was established to assess the effectiveness of the teaching system. The assessment strategy incorporated 3 primary components: performance assessment through standardized practical tests, learning experience evaluation via structured questionnaires, and comparative cost analysis of resource use. Key evaluation indicators encompassed experimental operation proficiency, theoretical knowledge mastery, student satisfaction levels, teaching resource efficiency, and safety protocol compliance. This multidimensional assessment approach enabled thorough evaluation of both learning outcomes and teaching effectiveness.

### Statistical Analysis

The collected data underwent rigorous statistical analysis using SPSS (version 25.0; SPSS Inc). Statistical methods included independent 2-tailed *t* tests for comparing group differences in continuous variables and *χ*^2^ tests for analyzing categorical data. All statistical analyses were conducted with a significance level set at *P*<.05. This analytical approach ensured robust evaluation of the teaching system’s effectiveness while maintaining scientific rigor in data interpretation.

### Ethical Considerations

This study was approved by the ethics committee of Chongqing Medical University (approval number 2023027). The study was conducted in accordance with local legislation and institutional requirements. We obtained informed consent from participants through an online questionnaire system. Participants were provided with a research information statement detailing the study’s purpose, participation requirements, potential risks and benefits, data protection measures, and a declaration that participation was voluntary. Participants were required to confirm they had read and understood this information and agreed to participate before being included in the study. No financial or material compensation was provided to participants. All data were fully anonymized prior to analysis.

## Results

### Virtual Laboratory System Development

**Figure 2.**
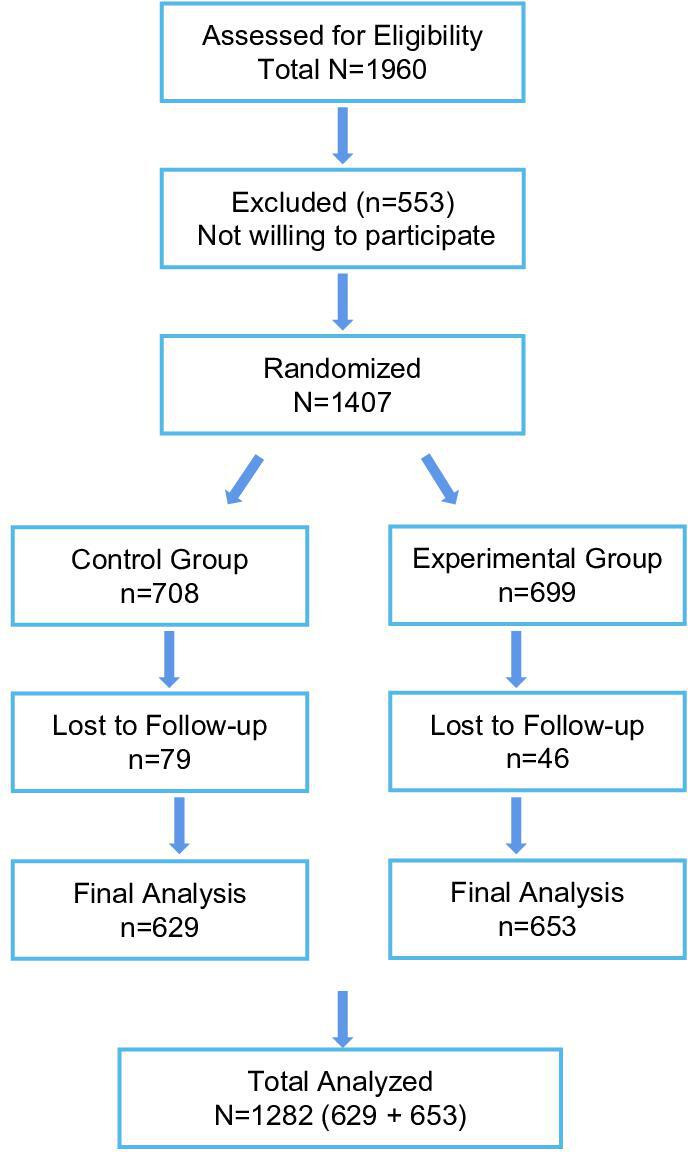
CONSORT (Consolidated Standards of Reporting Trials) flow chart of participation.

The developed virtual laboratory system (Multimedia Appendix 1) successfully integrated 3 core modules: knowledge review, a learning module, and an assessment module. The experimental procedure flow (Multimedia Appendix 2) was effectively translated into the virtual environment. The 3-module design received high approval from 1214 of 1282 (94.7%) participants across all medical specialties (Multimedia Appendix 3). Figure 2 shows the CONSORT (Consolidated Standards of Reporting Trials) diagram of participation flow.

### Learning Performance Analysis

Comparative analysis revealed significant differences in test scores between the experimental and control groups (Table 1). The experimental group demonstrated higher performance across multiple medical fields, with particularly notable improvements in clinical medicine, nursing, traditional Chinese pharmacology, and the science of acupuncture and moxibustion (overall *P*<.001).

**Table 1.** Experiment test scores of students from different majors.

Subject	Control group score, mean (SD)	Experiment group score, mean (SD)	*P* value
Clinical medicine	68.34 (17.23)	89.88 (13.09)	<.001
Traditional Chinese medicine	60.54 (17.01)	67.97 (13.14)	.02
Pediatrics	76.29 (18.83)	84.84 (15.26)	.003
Nursing	64.25 (12.56)	73.11 (16.82)	<.001
Medical imaging	70.39 (19.39)	79.94 (18.50)	.01
Preventive medicine	68.83 (15.13)	78.56 (17.72)	.01
Clinical pharmacy	69.41 (21.12)	86.53 (16.05)	.003
Science of acupuncture and moxibustion	60.11 (11.32)	71.47 (15.66)	<.001
Traditional Chinese pharmacology	65.33 (11.28)	82.96 (14.16)	<.001
Overall	67.45 (16.81)	80.82 (17.10)	<.001

### System Advantages Analysis

Student perception analysis (Table 2) highlighted key advantages of the virtual system: 90.8% of students reported enhanced safety, 79.6% appreciated the unlimited repetition capability, and 74.6% mentioned cost reduction. The standardized experimental conditions (61.6%) and personalized teaching features (50.6%) also received positive recognition.

**Table 2.** Distribution of responses among medical students (n=1282) on key advantages of virtual simulation experiments.

Option	Responses, n (%)
Enhanced safety	1164 (90.8)
Standardized experimental conditions	790 (61.6)
Lower costs	957 (74.6)
Unlimited repetition	1020 (79.6)
Simulating abnormal situations	701 (54.7)
Personalized teaching	649 (50.6)
Intelligent assessment system	607 (47.3)

### Interface Design and Implementation

The interface design evaluation (Multimedia Appendix 4) showed that 658 of 1282 (51.3%) students found it well designed and clear. Regarding difficulty levels (Multimedia Appendix 5), 1168 (91.1%) students found the experiment appropriately challenging, and 1156 (90.2%) students reported that the first-person perspective enhanced their learning experience (Table 3), with the 3-module design receiving high approval (Multimedia Appendix 3).

**Table 3.** First-person perspective: impact on biosafety level 2 lab experience and understanding of microbial techniques among medical students (n=1282).

Medical specialty	Significantly enhanced, n (%)	Little impact, n (%)	Uncertain, n (%)
Total	1156 (90.2)	62 (4.8)	64 (5.0)
Clinical medicine	340 (87.9)	21 (5.4)	26 (6.7)
Traditional Chinese medicine	89 (88.1)	8 (7.9)	4 (4.0)
Pediatrics	136 (90.1)	7 (4.6)	8 (5.3)
Nursing	243 (93.1)	10 (3.8)	8 (3.1)
Medical imaging	98 (89.9)	5 (4.6)	6 (5.5)
Preventive medicine	70 (93.3)	3 (4.0)	2 (2.7)
Clinical pharmacy	47 (88.7)	2 (3.8)	4 (7.5)
Science of acupuncture and moxibustion	92 (92.9)	2 (2.0)	5 (5.1)
Traditional Chinese pharmacology	41 (89.1)	4 (8.7)	1 (2.2)

### Learning Outcomes and Satisfaction

The results showed that 1268 of 1282 (98.9%) students reported improved comprehension (Table 4). Student acceptance data (Table 5) indicated that 1145 (89.3%) were willing to adopt virtual simulation as a supplementary learning tool. Overall satisfaction (Table 6) reached 97.2%, with 39.6% being “very satisfied” and 57.6% “generally satisfied.”

**Table 4.** Enhancement of knowledge and understanding through virtual simulation experiments among medical students (n=1282).

Medical specialty	Significantly deepened understanding, n (%)	Somewhat helpful, n (%)	Little to no help, n (%)
Total	383 (29.9)	885 (69.0)	14 (1.1)
Clinical medicine	109 (28.2)	269 (69.5)	9 (2.3)
Traditional Chinese medicine	26 (25.7)	75 (74.3)	0 (0.0)
Pediatrics	47 (31.1)	103 (68.2)	1 (0.7)
Nursing	78 (29.9)	183 (70.1)	0 (0.0)
Medical imaging	32 (29.4)	77 (70.6)	0 (0.0)
Preventive medicine	21 (28.0)	54 (72.0)	0 (0.0)
Clinical pharmacy	21 (39.6)	30 (56.6)	2 (3.8)
Science of acupuncture and moxibustion	37 (37.4)	60 (60.6)	2 (2.0)
Traditional Chinese pharmacology	12 (26.1)	34 (73.9)	0 (0.0)

**Table 5.** Survey on willingness to use virtual simulation experiments as a supplement to pathogenic biology experiments among medical students (n=1282).

Medical specialty	Willing, n (%)	Unwilling, n (%)	Indifferent, n (%)
Total	1145 (89.3)	73 (5.7)	64 (5.0)
Clinical medicine	341 (88.1)	26 (6.7)	20 (5.2)
Traditional Chinese medicine	87 (86.1)	10 (9.9)	4 (4.0)
Pediatrics	138 (91.4)	4 (2.6)	9 (6.0)
Nursing	235 (90.0)	17 (6.5)	9 (3.5)
Medical imaging	97 (89.0)	6 (5.5)	6 (5.5)
Preventive medicine	70 (93.3)	1 (1.3)	4 (5.3)
Clinical pharmacy	49 (92.5)	3 (5.7)	1 (1.9)
Science of acupuncture and moxibustion	87 (87.9)	5 (5.1)	7 (7.1)
Traditional Chinese pharmacology	41 (89.1)	1 (2.2)	4 (8.7)

**Table 6.** Overall satisfaction assessment of the virtual simulation experiment among medical students (n=1282).

Medical specialty	Highly satisfied, n (%)	Generally satisfied, n (%)	Dissatisfied, n (%)
Total	508 (39.6)	738 (57.6)	36 (2.8)
Clinic medicine	164 (42.4)	209 (54.0)	14 (3.6)
Traditional Chinese medicine	29 (28.7)	70 (69.3)	2 (2.0)
Pediatrics	62 (41.1)	84 (55.6)	5 (3.3)
Nursing	94 (36.0)	161 (61.7)	6 (2.3)
Medical imaging	41 (37.6)	66 (60.6)	2 (1.8)
Preventive medicine	31 (41.3)	43 (57.3)	1 (1.3)
Clinical pharmacy	23 (43.4)	27 (50.9)	3 (5.7)
Science of acupuncture and moxibustion	43 (43.4)	53 (53.5)	3 (3.0)
Traditional Chinese pharmacology	21 (45.7)	25 (54.3)	0 (0.0)

## Discussion

### Principal Findings

Virtual simulation technology offers innovative and effective solutions for teaching related to laboratory experiments [20-23]. Our program was developed to address a critical training gap identified in institutional needs assessments: over 80% of students sought enhanced training in pathogenic bacteria, yet 90% of faculty opposed live cocci experiments due to biosafety concerns.

The 3-module design (the knowledge review, learning module, and assessment module) successfully reconciled this “must-learn vs cannot-do” paradox, achieving 94.7% student approval. This structure aligns with cognitive learning theory [20] while specifically addressing the staged requirements of microbiology experiments [21]. Quantitative assessments confirmed significant improvements across specialties (*P*<.05), including a 98.9% enhancement in theoretical understanding and 90.8% increase in safety awareness—effectively overcoming key limitations of traditional teaching through (1) standardized experimental procedures, (2) 60% cost reduction over 5 years, and (3) integrated BSL-2 safety training.

These findings are corroborated by prior research [11,24-26], particularly regarding virtual laboratories’ advantages in safety and accessibility. The program’s cost-effectiveness and scalability (serving large student populations with easy updates) will allow the further optimization of teaching models [7-9] and expansion of remote education possibilities.

Nevertheless, real experiments retain irreplaceable advantages in cultivating practical operational skills and sensory experiences [27]. Feedback from 1.1% of students indicating limited assistance from virtual experiments highlights the persistent limitations in substituting for hands-on operations in real environments [4-6]. Consequently, virtual simulation experiments and real experiments each possess distinct advantages [13-15,28]. The optimal teaching strategy should therefore involve an organic integration of both approaches [16-18,29].

Based on tracking student feedback, we identified the main issues with the virtual experiment system as lag, lack of speed adjustment options, poor learning module integration, incomplete results analysis, and inadequate error simulation compared to real experiments. To address these problems, we developed a series of optimization strategies. First, server performance should be enhanced and the program’s code should be optimized to resolve lag issues. Second, the introduction of an intelligent time management system would allow students to adjust the experiment’s progress. In terms of instructional design, the learning modules could be improved by increasing interactive guidance and feedback. Simultaneously, incorporating data visualization tools would strengthen results analysis, and refining the simulation fidelity would allow it to better reflect real experimental conditions. Additionally, we designed a multidimensional evaluation system to continuously collect and analyze student feedback for iterative system optimization. Lastly, it is important to strengthen teacher training, which is crucial for guiding student learning through virtual experiments. Through comprehensive implementation of these strategies [30], we expect to significantly enhance the technical implementation, instructional design, and learning experience of virtual experiments, thereby substantially improving their educational effectiveness.

To ensure analytical rigor, this study used a complete case analysis method, including only data from students who completed both tests and questionnaires. While this approach enhanced data quality and analytical consistency, it also reduced the effective sample size, potentially limiting the generalizability of the results. Future research might consider advanced techniques such as multiple imputation to handle missing data and extending the scope of the study to different educational levels to enhance its representativeness. Subsequent studies should adopt a mixed methods approach, integrating quantitative analysis with qualitative research, and use a longitudinal design to assess the long-term educational effectiveness of virtual experiments. These research directions would deepen understanding of virtual experiments’ educational effectiveness, promote teaching innovation, and provide crucial guidance for future education practices.

### Conclusion

The virtual simulation experiment system described here serves as an effective complement to real experiments, offering significant advantages in terms of safety, repeatability, and standardization. Our study demonstrates its effectiveness through improved student performance and a high satisfaction rate (97.2%). The 3-module design effectively enhanced students’ understanding (98.9% reporting improvement) and engagement with the experimental procedures. While virtual experiments show distinct advantages in visualization and allow repeated practice, they work best when integrated with traditional hands-on experiments to provide a comprehensive learning experience. This combination leverages the strengths of both approaches—the safety and flexibility of virtual simulation with the irreplaceable tactile experience of real laboratory practice. Future development should focus on technical optimization and enhanced integration with traditional teaching methods to further improve educational outcomes.

## Supplementary material

10.2196/72495 Multimedia Appendix 1 Ideas for identifying pathogenic cocci.

10.2196/72495 Multimedia Appendix 2 Virtual experiment process.

10.2196/72495 Multimedia Appendix 3 Evaluation of virtual experiment's 3-module design: review, learning, and assessment.

10.2196/72495 Multimedia Appendix 4 Evaluation of the virtual simulation experiment interface design and usability.

10.2196/72495 Multimedia Appendix 5 Perceived difficulty levels of the virtual simulation experiments.

10.2196/72495
Checklist 1
CONSORT (Consolidated Standards of Reporting Trials) checklist.

## Abbreviations

BSL: biosafety level
CONSORT: Consolidated Standards of Reporting Trials
