# Supplementary figures and images for: A Large-Scale Multispecialty Evaluation of Web-Based Simulation in Medical Microbiology Laboratory Education: Randomized Controlled Trial
Source: JMIR Med Educ. 2025 Jul 30;11:e72495. doi: 10.2196/72495 (PMC12310184; doi:10.2196/72495)

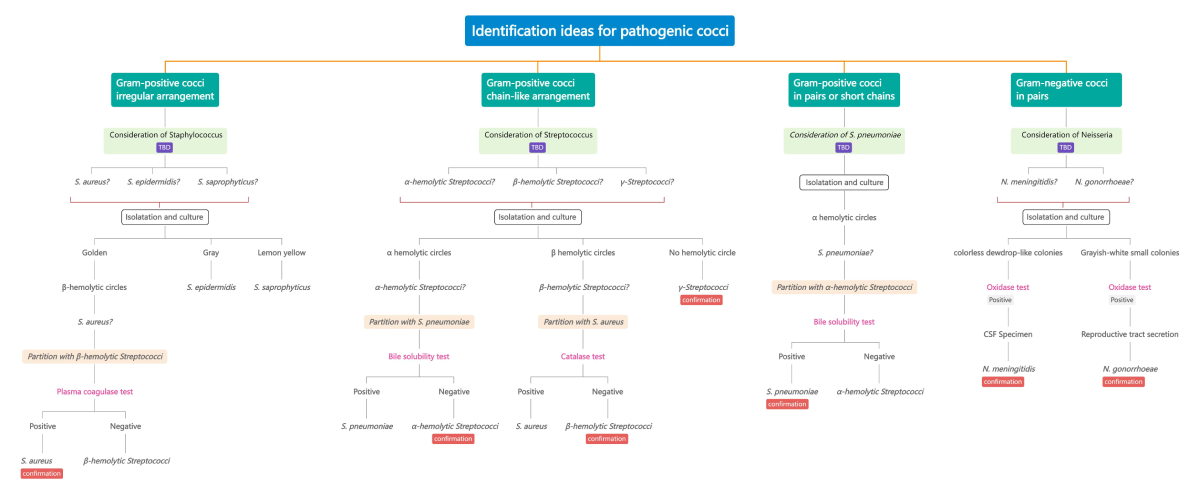

Supplement: Multimedia Appendix 1 [file mededu-v11-e72495-s001.png]

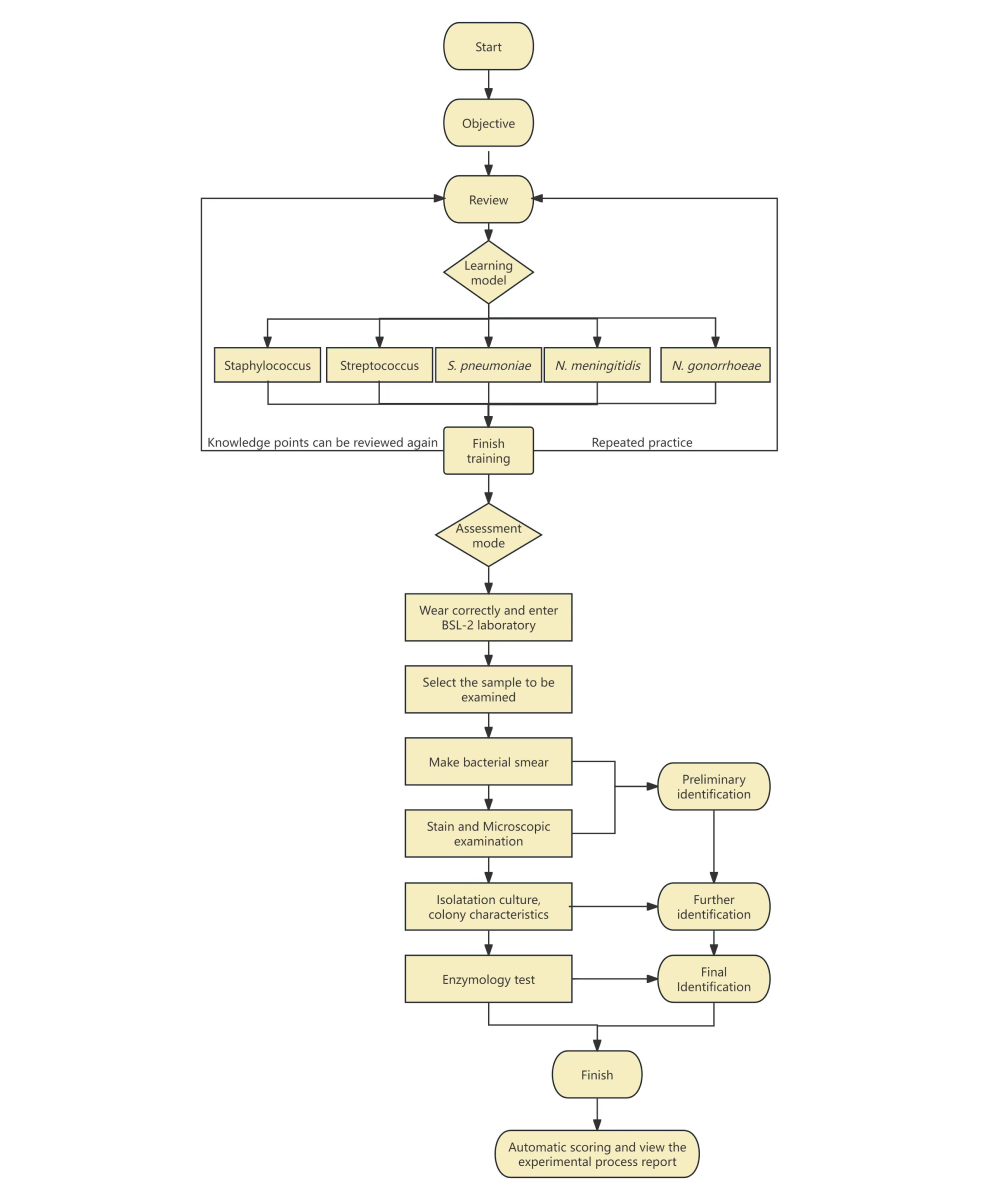

Supplement: Multimedia Appendix 2 [file mededu-v11-e72495-s002.png]
